# An Appreciation of Mr. W. G. Carnt

**Published:** 1915-09-11

**Authors:** Keith D. Young

**Affiliations:** 17 Southampton Street, Bloomsbury, London, W.C.


### The Editor's Letter-Box.

### An Appreciation of Mr. W. G. Carnt.

*To the Editor of* The Hospital.

Sir,—I shall be glad if you will allow me to add briefly to the excellent appreciation of Mr. Carnt and his work which appears in your (this week's) issue.

My late partner, Mr. Henry Hall, and I worked in close co-operation with Mr. Carnt during the time when we were designing the Derbyshire Royal Infirmary; and I could not speak too highly of the untiring, loyal, and altogether invaluable help he gave us then, and in the succeeding years when additions had to be devised.

The Derby appointment was Mr. Carnt's first experience of hospital work; but if he had been brought up to the work he could not have done better than he did.

In the midst of all the strenuous work that fell to his lot at Manchester he never failed to respond generously to any appeal made to him for advice or information on matters connected with hospital work.

That his retirement is due to a physical breakdown is a fact that will cause deep and heartfelt sorrow to his many friends, among whom I am proud to number myself.—Yours faithfully, Keith D. Young.

17 Southampton Street, Bloomsbury,
London, W.C., September 6, 1915.

